# Do Parents Still Matter Regarding Adolescents’ Alcohol Drinking? Experience from South Africa

**DOI:** 10.3390/ijerph9010110

**Published:** 2012-01-04

**Authors:** Muhammad Hoque, Shanaz Ghuman

**Affiliations:** 1 Department of Public Health, School of Health Care Sciences, University of Limpopo (Medunsa Campus), Pretoria 0204, South Africa; 2 Department of Environmental Health, Mangosuthu University of Technology, P.O. Box 12363, Jacobs 4026, Durban, South Africa; Email: shanaz@mut.ac.za

**Keywords:** alcohol, consumption, adolescent, parent, South Africa

## Abstract

The purpose of this cross-sectional study was to improve our understanding of adolescents’ perceptions of parental practices relating to their (adolescents’) alcohol use. A total of 704 students were conveniently selected and completed self-administered questionnaires. More than half (54%) of the adolescents reported that they had consumed alcohol at some time in their life. Parental marital status was significantly associated with whether adolescents ever consumed alcohol or not (*p* < 0.05). A large number of mothers/female guardians (66.3%) and fathers/male guardians (69.3%) did not allow alcohol use at home. More mothers (54.6%) and fathers (65.3%) were not aware of their adolescents’ alcohol consumption (*p* < 0.05). Adolescents were more likely to use alcohol when they reported that they had often seen either their father or mother drunk or both (*p* < 0.05). There were also significant associations between parents’ views against alcohol use and their adolescents’ alcohol use (*p* < 0.05). Prevalence of alcohol uptake was quite high among these adolescents. Compulsory parenting programmes and skills development should be practiced by education, health, cultural and religious groups. Parents should be motivated to delay the age at which their children are initiated into alcohol use and be provided with guidance on how to counteract social pressures.

## 1. Introduction

Globally, alcohol consumption has increased in recent decades, particularly in developing countries [1], and alcohol use now ranks fifth amongst the leading causes of death worldwide [1]. The rise in alcohol consumption in developing countries provides ample cause for concern over the possible advent of a matching rise in alcohol-related problems in those regions of the world most at risk to substance abuse [1]. Underage drinking is a serious public health issue. Youths who start drinking early are at an elevated risk of using alcohol and hard drug abuse later in life [2,3]. The WHO has observed the drop in age of onset of alcohol use by children and adolescents as a problem and has suggested using effective strategies to delay the onset of alcohol use in its member states [4].

In a South African study it was reported that 25% of Grade 8–10 students had used tobacco; a third had used alcohol and 13% had used drugs as early as the age of 13 years [5]. In another study that was conducted in 28 high schools in Durban, which is the third largest city and the largest city in the South African province of KwaZulu-Natal, revealed that 45.4% of male and 25.5% of female students in grade 11 reported past year alcohol use [6]. Surveys from a study at three sites in South Africa reported high levels of alcohol misuse among high school students, with alcohol being the most common substance of abuse [7]. Alcohol constitutes the second most frequently reported primary substance of abuse among adolescent patients in Durban [7]. The study also revealed that of 35 state schools in the year 2000, 40% of students reported drinking to intoxication occasionally during the course of a typical month. The prevalence of binge drinking increases with age for both genders which is in keeping with international findings on alcohol use trends among adolescents [8].

Positive parental influences through effective monitoring, support, close child-parent relationships and trust have been found to protect against negative peer pressure for health risk behaviours [9,10]. Studies have shown that confident parents, who legitimately have the right to impact on their child’s substance use behaviours, are the ones who are more capable of exerting specific anti-substance use socialisation practices [11]. Such parents are also successful at facilitating positive adolescent behaviours [9,12]. Parental disapproval of risky behaviours and substance use, and successful parent-child communication, is related to lower levels of adolescent risk behaviours [13,14]. Parents adjust the manner in which they communicate about risky behaviour according to the gender of the adolescent [15]. In a study it was reported that mothers rather than fathers play a pivotal role in communicating with their children and girls seem to receive more information than boys regarding risk behaviours [16]. These authors concluded that parental behaviours such as negative socialisation skills and practices (like being drunk in the presence of their children) are significant precursors of disruptive behaviour, vulnerability and succumbing to peer pressure and substance use by their adolescent children.

There is consistent evidence that amongst middle- and high school aged adolescents; parental practices of monitoring and communication are protective measures against alcohol and drug use [17,18]. National researchers have emphasised that South African youth have increasing levels of health risk behaviours which need to be urgently addressed [7,19]. While adolescents have been subjected to various risk prevention interventions the need to offer parental guidance programmes remains an urgent priority. There has been very little research on parenting practices which may help identify parenting styles among South African parents. Therefore, this study is an explorative study aiming to improve our understanding of adolescents’ perceptions of parental practices relating to adolescents’ alcohol use. 

## 2. Methods

### 2.1. Research Design and Sampling

A cross-sectional survey was conducted during 2007. All adolescents, 16 to 18 years old from five high schools in the Emawaleni District of KwaZulu-Natal (KZN) were eligible to participate in the study. One thousand two hundred and twenty seven adolescents were approached to participate in the study. Only the 704 learners, who had written consent from their parents/guardian for participation in the study, completed a self-administered questionnaire.

### 2.2. Research Instrument

A self-administered questionnaire in English was developed as it is the medium of instruction in secondary schools. However, English is not the home language of many learners and therefore elementary English was used to ensure comprehension. The questionnaire was pre-tested for its appropriateness. Due to the fact that this was an exploratory study, a pilot study was primarily done among 90 adolescents from a high school to refine the questionnaire. The questionnaire contained items related to socio-demographics of the adolescent and included age, gender, race, home language, as well as parent details such as educational background and marital status. Questions on adolescents’ alcohol use or non use, and parental alcohol use and socialisation behaviours were included. Adolescents’ perceptions of their parents’ alcohol socialisation behaviours as well as the house rules regarding alcohol were investigated. A four-point Likert Scale was used for questions which focused on house rules regarding alcohol use and ranged from “definitely true” to “definitely not true”, for example: “I am not allowed to drink alcohol at home or anywhere else”. Two questions were open-ended and unstructured which provided the adolescent an opportunity to freely respond to the questions which measured their perceptions of safe and risky drinking behaviours. These questions were “What do you think is the most serious risk associated with drinking alcohol?” and “What do you think safe drinking means?” A question with a “yes” or “no” response assessing parental communication with regards to alcohol use was used to determine whether parents spoke to their adolescent about alcohol use. Adolescent perceptions of parental practice such as communication, trust, monitoring and attitudes towards alcohol use were included. 

### 2.3. Ethical Clearance

Ethical clearance was obtained from the Biomedical Research Ethics Committee of the Nelson R Mandela School of Medicine, University of KwaZulu-Natal, South Africa. Permission was obtained from the Provincial Department of Education and Principals of participating schools. An information sheet about the aims of the study was sent home to the learners’ parents or guardians to obtain written informed consent for the participation of their children in the study. Ethical issues such as voluntary participation, confidentiality and anonymity of the data were explained to parents/guardian as well as to the learners. Written informed consent was also obtained from the learners prior to the completion of the questionnaires. All questionnaires were completed anonymously. 

### 2.4. Data Collection

The information and consent documents were sealed in envelopes and sent to 1227 parents of adolescents. Eight hundred and seven parents responded positively (66%). The signature of one parent or legal guardian was required on the consent form for permission and this was returned to the school by the learner after the parent or legal guardian had read and removed the information letter. Only those learners, who brought the consent document signed by their parent or legal guardian, back to school, were eligible to participate in the study. The researcher conducted the survey in an appropriate venue at each of the schools’ hall with the permission of the principals and educators at each school. Educators were not involved in this process. The questionnaires were completed within 45 minutes and collected by the researcher. Only 704 completed (57%) questionnaires remained in the possession of the main researcher. The remaining 9% either withdrew consent, did not attend on the day of the survey and a few questionnaires could not be used due to incomplete information. Data were collected during January to April 2007.

### 2.5. Data Analysis

Data were entered into a Microsoft Excel 2003 spreadsheet and imported into SPSS 17.0.1 for analysis. The results of participants’ were summarized using descriptive summary measures: expressed as mean (SD) or median (range) for continuous variables and percent for categorical variables. Chi-square tests were used to find associations between drinking alcohol and socio-demographic variables. All statistical tests were performed using two-sided tests at the 0.05 level of significance. *P*-values are reported to three decimal places with values less than 0.001 reported as <0.001.

## 3. Results

One thousand two hundred and twenty seven adolescents were approached to participate in the study. Only 704 learners participated in the study, with a response rate of 57.4%. The demographic information of the participants is shown in Table 1. The participating adolescents were between the ages of 16 and 18 years. Of the 704 participants, 59.8% were female. In terms of race, less than half (46.2%) were African, followed by Indian and White. English and Zulu were the most common home languages of the participants.

**Table 1:** Demographic characteristics of study participants from public high schools in the Emawaleni District (*N* = 704).

Characteristic	Frequency	Percentage
**Age**		
16 years	300	42.6
17 years	292	41.5
18 years	112	15.9
**Gender**		
Male	283	40.2
Female	421	59.8
**Grade**		
11	374	53.1
12	330	46.9
**Race**		
Asian/Indian	196	27.8
Black/African	325	46.2
White	183	26.0
**Home language**		
Afrikaans	64	9.1
English	317	45.0
Zulu	308	43.8
Other	15	2.1

Regarding ever alcohol use, more than half (54%) of the adolescents reported that they had consumed alcohol at some time in their life. The main caregivers of a large number of the adolescent participants were both parents (62.6%) who lived with them at their home and 29% who lived with a guardian (Table 2). A large percentage (57.8%) of adolescents’ mothers/female guardians had a tertiary qualification, and 21.4% had a Matric qualification. Fathers/male guardians’ educational status followed a similar pattern with 60.1% having a tertiary qualification and 22.2% having matriculated. The majority of the adolescents’ parents/guardians were married (61.1%). Chi-square test of association indicated that guardian’s marital status was significantly associated with adolescents ever drank or not (*p* < 0.05) as 81% of the students whose parents were divorced compared to 51% students whose parents were married and living together ever consumed alcohol but was not associated with whoever takes care of them or their education level (*p* > 0.05).

**Table 2:** Caregivers socio-demographic information in relation to adolescents alcohol drinking.

Variables	Ever Drank Alcohol		*p* value
	No	Yes	
**Who takes care of you at home?**			
Father & mother	197	244	0.279
Mother/female only	104	101	
Father/male only	24	34	
**Education**			
No education	11	14	0.105
Primary	12	15	
Secondary	39	37	
Matric	104	89	
Tertiary	93	138	
Postgraduate	66	86	
**Marital status**			
Married living together	196	234	<0.001
Married not living together	15	18	
Unmarried living together	6	7	
Unmarried not living together/single	68	46	
Divorced	11	49	
Other	29	25	

Table 3 reports adolescents’ perception regarding their parents’ awareness on adolescent alcohol use. Adolescents reported that 26.7% of mothers/female guardians and 22.6% of fathers/male guardians were aware of their alcohol use. A large number of mothers/female guardians (66.3%) and fathers/male guardians (69.3%) did not allow alcohol use at home. More mothers (54.6%) and fathers (65.3%) were not aware of their adolescents’ alcohol drinking (*p* < 0.05). Also significantly more adolescents’ drank alcohol whose parents did not allow them to drink alcohol (*p* < 0.05).

**Table 3:** Adolescents’ perception on parental awareness and acceptance on adolescent alcohol use.

Variables		Ever Drank Alcohol		*p* value
		No	Yes	
Mother know about their adolescent alcohol drinking	Yes	16 (4.9)	172 (45.4) *	<0.001
	No	65 (20.0)	176 (46.4)	
	Don’t Know	244 (75.1)	31 (8.2)	
Father know about their adolescent alcohol drinking	Yes	16 (4.9)	143 (37.7)	<0.001
	No	104 (32.0)	210 (55.4)	
	Don’t Know	205 (63.1)	26 (6.9)	
Mother allows drinking alcohol	Yes	22 (6.8)	130 (34.3)	<0.001
	No	268 (82.5)	199 (52.5)	
	Don’t know	35 (10.8)	50 (13.2)	
Father allows drinking alcohol	Yes	16 (4.9)	120 (31.7)	<0.001
	No	277 (85.2)	211 (55.7)	
	Don’t Know	32 (9.8)	48 (12.7)	
Mother allow drinking alcohol with friends	Yes	8 (2.5)	115 (30.3)	<0.001
	No	292 (89.8)	209 (55.1)	
	Don’t know	25 (7.7)	55 (14.5)	
Father allows drinking alcohol with friends	Yes	8 (2.5)	94 (24.8)	<0.001
	No	286 (88.0)	228 (60.2)	
	Don’t know	31 (9.5)	57 (15.0)	

* Column percentage.

Table 4 shows association between adolescents’ parents’ alcohol uses and house rules regarding alcohol uses and adolescents’ alcohol use. There were significant associations between adolescents’ parents’ alcohol use and their adolescents’ alcohol use (*p* < 0.05). Adolescents were more likely to use alcohol when they reported that they had often seen either their father or mother drunk or both (*p* < 0.05). There were also significant associations between parents view against alcohol use and their adolescents’ alcohol use as less adolescents ever drank alcohol whose fathers were against alcohol use but more adolescents used alcohol whose mothers were against alcohol use (*p* < 0.05). 

**Table 4:** Adolescent’s perception regarding their parents’ alcohol uses and house rules regarding alcohol uses.

Variables		Adolescent Ever Drank Alcohol		*p* value
		No	Yes	
Mother drinks alcohol	Often	7 (2.2)	31 (8.2) *	<0.001
	Sometimes	28 (8.6)	81 (21.4)	
	Never	290 (89.2)	267 (70.4)	
Father drinks alcohol	Often	35 (10.8)	87 (23.0)	<0.001
	Sometimes	85 (26.2)	124 (32.7)	
	Never	205 (63.1)	168 (44.3)	
In our house there are very clear rules about drinking alcohol	Definitely true	183 (56.3)	131 (34.6)	<0.001
	True	92 (28.3)	156 (41.2)	
	Not true	34 (10.5)	66 (17.4)	
	Definitely not true	16 (4.9)	26 (6.9)	
Adults are allowed to drink alcohol at our home but not children	Definitely true	73 (22.5)	71 (18.7)	<0.001
	True	82 (25.2)	137 (36.1)	
	Not true	73 (22.5)	105 (27.7)	
	Definitely not true	97 (29.8)	66 (17.4)	
My father is against the use of alcohol	Definitely true	112 (34.5)	77 (20.3)	<0.001
	True	65 (20.0)	61 (16.1)	
	Not true	111 (34.2)	161 (42.5)	
	Definitely not true	37 (11.4)	80 (21.1)	
My mother is against the use of alcohol	Definitely true	191 (58.8)	145 (38.3)	<0.001
	True	61 (18.8)	70 (18.5)	
	Not true	49 (15.1)	121 (31.9)	
	Definitely not true	24 (7.4)	43 (11.3)	
I am not allowed to drink alcohol at home or anywhere else	Definitely true	216 (66.5)	115 (30.3)	<0.001
	True	60 (18.5)	88 (23.2)	
	Not true	33 (10.2)	123 (32.5)	
	Definitely not true	16 (4.9)	53 (14.0)	
No one else is allowed to drink alcohol in our house	Definitely true	126 (38.8)	69 (18.2)	<0.001
	True	52 (16.0)	43 (11.3)	
	Not true	100 (30.8)	147 (38.8)	
	Definitely not true	47 (14.5)	120 (31.7)	

* Column percentage.

From the bivariate analysis, all the variables that were significantly associated with alcohol use were entered into backward stepwise logistic regression model. Table 5 shows the final model of the regression analysis. The results indicated that having clear house rules was a protective factor for alcohol use among the high school students. The model fitted adequately (Chi-square = 433.929, *p* < 0.001) and overall prediction was quite good as Nagelkerke *R* Square = 0.615.

**Table 5:** Backward stepwise logistic regression output for ever alcohol use among the students.

Variables ^a^	B	S.E.	*p*-value	Odds Ratio (OR)	95% C.I. for OR	
					Lower	Upper
Mother know (Yes) *	3.663	0.365	<0.001	38.988	19.047	79.807
Mother know (No)	3.222	0.261	<0.001	25.068	15.039	41.785
Father allows drinking (Yes) *	0.727	0.498	0.144	2.069	0.780	5.486
Father allows drinking (No)	−0.579	0.379	0.127	0.561	0.267	1.178
Mother allow drinking with friends (Yes) *	0.995	0.559	0.075	2.706	0.905	8.089
Mother allow drinking with friends (No)	−0.287	0.379	0.449	0.750	0.357	1.578
Clear house rules (Definitely True) **	−1.371	0.498	0.006	0.254	0.096	0.674
Clear house rules (True)	−0.653	0.501	0.192	0.520	0.195	1.389
Clear house rules (Not true)	−1.128	0.563	0.045	0.324	0.107	0.977
Constant	−0.525	0.574	0.360	0.591		

^a^ Variable(s) entered on step 1: marital status, mother knows, father knows, mother allows drinking, father allows drinking, mother allows drinking with friends, father allows drinking with friends, mother drunk, father drunk, clear house rules, adults not children drink at home, father against drinking, mother against drinking, no drinking at home, no one drinks in house; Omnibus Tests of Model Coefficients: Chi-square = 433.929, *p* < 0.001; Model Summary: −2 Log likelihood = 537.876, Nagelkerke R Square = 0.615; * Do not know as reference group, ** Definitely not true as reference group.

**Figure 1:**
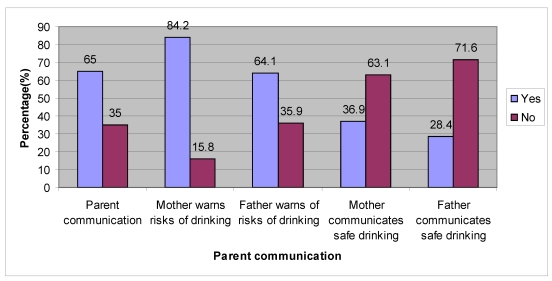
Parental communication with adolescents regarding risky and safe alcohol use.

In terms of communication, 65% of adolescents’ parents did communicate with their adolescents. With regard to alcohol use, 84% of mothers and 64% of fathers were found to communicate with the adolescents regarding the risks of using alcohol. In terms of safe drinking habits, 28% of fathers and 37% of mothers communicated with their children regarding safe drinking practices (Figure 1).

## 4. Discussion

The study investigated the importance of parental influence on adolescent alcohol use and the associations between various demographic variables, parental practices as perceived by adolescents and alcohol use amongst 16 to 18 year old high school learners. More than half (54%) of the adolescents had consumed alcohol at some time in their life. Parental awareness, uses of alcohol, and views on alcohol were significantly associated with their adolescents ever alcohol use.

Adolescents’ alcohol uses in one-month period were investigated. More than half of the adolescents had consumed alcohol at some time in their life. This rate is higher than that of a study conducted among rural high school students in Limpopo Province in South Africa (6.4%) [20]. Another study reported that current use of alcohol among various samples among adolescents assessed from 1993 to 2006 found a range of current alcohol use ranging from 21.5% to 62% [21]. Adolescents of 12–20 years reported that they had used alcohol in a one month period (44.9%) and 28.8% admitted to binge-drinking. Another study concluded that among high school youth who drank alcohol, binge-drinking was strongly associated with a wide range of other health risk behaviours [22]. In a South African study it was revealed that 25% of Grade 8–10 learners had used tobacco; a third had used alcohol and 13% had used drugs as early as the age of 13 years [5]. These prevalence levels amongst adolescents allude to determining protective and resiliency factors that will prevent or reduce the intake of substance use. The challenge will remain to decrease the current rates and to maintain low prevalence rates as these adolescents mature into young adulthood.

Mothers’ occupation had little effect on parenting styles as they retained the traditional role of housewife despite their educational level. This is different from a study where parent educational levels showed that higher levels of education are related to less harsh parenting styles amongst mothers [23].

In terms of the parental marital status the findings revealed that 82% of adolescents who had used alcohol came from a family structure where parents were divorced or had separated. These findings concurred with the literature presented. Adolescents who resided in one-parent households were more likely to participate in risk behaviours, such as using alcohol [24,25]. However, fifty-two percent of adolescents in one-parent households are 2.5 times more likely to report non-use of alcohol if they are actively involved in community activities [25]. Various studies report lower levels of alcohol use among adolescents in two-parent families than among adolescents in single-parent families [26,27]. 

This study found that a large number of mothers/female guardians (66.3%) and fathers/male guardians (69.3%) did not allow drinking alcohol at home. It was argued that when adolescents are not permitted to drink alcohol in the home, it has a protective effect on reducing the initiation age of alcohol use. They found that the age of 16 or 17 was usually when parents allowed their adolescents to drink alcohol at home [28].Parental disapproval also appeared to result in lowered adolescent drinking patterns [29]. 

The study found that twenty seven percent (27%) of mothers/female guardians and 23% of fathers/male guardians were aware of their adolescents’ drinking behaviours. As a significant difference between parental reports and adolescent consumption patterns it was be concluded that parents do not always know the level of alcohol consumed by their adolescents and tend to underestimate their children’s use of alcohol [28]. A Canadian study reported that of 854, 12 to 18-year-old adolescents, only 34% of parents were aware that their adolescents used alcohol [28]. Based on a series of quantitative and qualitative studies in Australia, we can argue that parents are more concerned with illegal drug use than with adolescent alcohol use [30]. 

Adolescents in this study reported that 17% of fathers drank often and that 5% of mothers often used alcohol. The majority of adolescents (66%) seemed to think that their mothers were against them using alcohol while 45% thought that their fathers were against their use of alcohol. Researchers have all shown significant positive relationships between youths who were less likely to use alcohol and having positive family relationships and peer role models [31]. A study of 13 to19-year-old adolescents pertaining to alcohol non-use and the availability of peer role models found that adolescents with positive family communication, good health practices and future aspirations were 1.5 to 2.5 times less likely to use alcohol than the others [25]. When parents use alcohol frequently, their adolescents have an increased likelihood of exposure to alcohol-related risk behaviours [28]. Wood and colleagues found that where parents were more permissive towards alcohol use, their adolescents were more likely to engage in heavy binge-drinking [32]. 

The participants in the study were selected through non-probability purposive sampling, as the learners who were able to participate were made available at each school’s convenience and thus could not be stipulated by the researcher. The study was carried out among only fluent English speaking adolescents from selected schools and the participants were not offered the opportunity to complete the questionnaire in their home language. The study area was limited to the Emawaleni District and only five of the twelve schools in this area had been selected. Also, adolescents may not have responded honestly to the questions, although studies have shown that adolescent reporting on their parents’ and own behaviours are far more accurate than parental reporting on their adolescents’ risk behaviours. Studies have shown that adolescents’ perceptions of parents are more accurate than parents’ perceptions of their adolescents [33,34]. Another limitation is that the study was conducted five years ago, but since no alcohol intervention program is in place in South Africa, the findings of this study might still be valid.

## 5. Conclusions

Prevalence of alcohol uptake was quite high among these adolescents and alcohol use was significantly associated with parental awareness, alcohol drinking and their views on their adolescents’ alcohol drinking. Compulsory parenting programmes and skills development should be practiced by education, health, cultural and religious groups. Interventions must be targeted before adolescence as well as throughout adolescence. This effort should not only help parents identify and adopt promising child management techniques, but should motivate attributes of monitoring, setting rules, interactive communication skills as well as stimulate a broader social context that enables parents to have the time to develop positive family relationships. Parents should be motivated to delay the age at which their children are initiated into alcohol use and be provided with guidance on how to counteract social pressures, especially from peers.
